# Engineered Silybin Nanoparticles Educe Efficient Control in Experimental Diabetes

**DOI:** 10.1371/journal.pone.0101818

**Published:** 2014-07-03

**Authors:** Suvadra Das, Partha Roy, Rajat Pal, Runa Ghosh Auddy, Abhay Sankar Chakraborti, Arup Mukherjee

**Affiliations:** 1 Department of Chemical Technology, University of Calcutta, Kolkata, West Bengal, India; 2 Department of Bio-Physics, Molecular Biology and Bioinformatics, University of Calcutta, Kolkata, West Bengal, India; 3 Centre for Research in Nanoscience and Nanotechnology, University of Calcutta, Kolkata, West Bengal, India; 4 Faculty of Technology (Pharmaceutical) University Malaysia Pahang (UMP), Pahang, Malaysia; University of Florida, United States of America

## Abstract

Silybin, is one imminent therapeutic for drug induced hepatotoxicity, human prostrate adenocarcinoma and other degenerative organ diseases. Recent evidences suggest that silybin influences gluconeogenesis pathways favorably and is beneficial in the treatment of type 1 and type 2 diabetes. The compound however is constrained due to solubility (0.4 mg/mL) and bioavailabilty limitations. Appropriate nanoparticle design for silybin in biocompatible polymers was thus proposed as a probable solution for therapeutic inadequacy. New surface engineered biopolymeric nanoparticles with high silybin encapsulation efficiency of 92.11% and zeta potential of +21 mV were designed. Both the pure compound and the nanoparticles were evaluated *in vivo* for the first time in experimental diabetic conditions. Animal health recovered substantially and the blood glucose levels came down to near normal values after 28 days treatment schedule with the engineered nanoparticles**.** Restoration from hyperglycemic damage condition was traced to serum insulin regeneration. Serum insulin recovered from the streptozotocin induced pancreatic damage levels of 0.17±0.01 µg/lit to 0.57±0.11 µg/lit after nanoparticle treatment. Significant reduction in glycated hemoglobin level, and restoration of liver glycogen content were some of the other interesting observations. Engineered silybin nanoparticle assisted recovery in diabetic conditions was reasoned due to improved silybin dissolution, passive transport in nanoscale, and restoration of antioxidant status.

## Introduction

Diabetes mellitus is a pathological condition which results in severe metabolic imbalances and is characterized by high blood glucose level, low blood insulin level or insensitivity of target organs to insulin. Prevalence of diabetes is growing globally at an alarming rate. The World Health Organization (WHO) projects that the disease will be the 7^th^ leading cause of death by the year 2030 [1]. Nearly 80% of diabetes deaths are currently reported from the highly populated countries including India, China and Thailand [2], [3]. Most of the synthetic antidiabetic drugs like sulphonylureas, biguanides, α-glucosidase inhibitors and thiazolidenes are associated with unwanted side effects and may cause significant diminution in glycemic responses [4].

Alternatively, different bioflavonoids from plant sources are regularly reported for control of postprandial glucose level. Quercetin for example, was observed to potentiate insulin release, enhance calcium uptake and facilitate regeneration of pancreatic islets. [5], [6]. However, flavonols and flavonoids are often associated with poor aqueous solubility and are easily metabolized in the gut and liver microsomes by enzyme systems such as catechol-O*-*methyltransferase (EC 2.1.1.6), phenol sulfotransferase (EC 2.8.2.1) or the UDP glucuronosyl transferases (EC 2.4.1.17) [7], [8]. Consistent delivery and smart dissolution of flavonoid candidate compounds are contemporary challenges that are studied widely to meet some of the precise therapeutic requirements. Application of current knowledge in apposite nanoparticle design appears one likely solution in that direction. Stevioside from *Stevia rebaudiana* leaves in Poly lactic acid (PLA) nanoparticles have been proposed recently as a new tool in control of diabetic conditions [9].

Silymarin (Sm) is one of the oldest traditional herbal medicines used to combat different organ disorders. Sm is predominantly composed of four flavonolignan isomers; silybin, isosilybin, silychristine and silydianin amongst which, silybin (Sb), is the key biologically active compound and constitutes 34% by mass of Sm [10], [11]. Hepatoprotective effects of Sb have been demonstrated repeatedly in humans and the most remarkable use of Sb was in the treatment of acute mushroom (*Amanita phalloides)* poisoning [12]. The compound is lowly toxic and exerts significant anti-carcinogenic and anti-inflammatory effects and its biomolecular mechanisms in different disease modifications have also been established [13], [14]. Sb has been very recently proposed to be beneficial in type 2 diabetes patients and a number of articles demonstrated decreases in both fasting and mean daily glucose, triglycerides and total cholesterol levels [15]–[17]. Aqueous extract of *Silybum marianum* rich in Sb exerts potent hypoglycemic and antihyperglycemic activities in both streptozotocin and alloxan-induced diabetic rats [18], [19]. Antidiabetic effect of Sb is attributed to inhibition of gluconeogenesis in the liver and decrease of glucose-6 phosphatase activity [20]. Sm acts as an agent for two way control against streptozotocin induced up regulation of cytochrome P 450 3A2 enzyme system and down regulation of glutathione peroxidase [21]. Liver and pancreas centric effects of Sb in control of diabetes are generally accepted but appropriate delivery design has remained a significant constraint in systematic evaluation and further applications. Sb is a low water soluble and low permeable biopharmaceutic class IV type compound that faces stipulated requirements in delivery design. Delivery development for Sb is therefore one urgent need to reap benefit in ailments such as diabetes. Flavonol centric antidiabetic design is also likely to provide a newer direction in management of one of the most rapidly progressing degenerative ailments in humankind.

Poly DL-lactide-co-glycolic acid (PLGA) is a safe; US FDA approved biopolymer for applications in nanopharmaceuticals. However, the biopolymer undergoes a degree of hydrolysis during its passage in GI tract [22]. Cationic modifications can enhance in appropriate pay load delivery. Polycationic modification with chitosan, a mucoadhesive permeability enhancer has been successful to that effect. Moreover, nanoparticle engineering with triblock pluronic biopolymers was earlier demonstrated by us to help in chitosan embossing and programmed drug dissolution [23]. Earlier systematic analysis and observations [24] in addition, incited us to exploit different techniques including nanoparticle engineering for loading a useful mass of the principle bioactive component Sb in PLGA based nanoparticles. Acetone is a widely accepted solvent for preparation of PLGA nanoparticles due to its miscibility with water in all proportions [25], [26]. Spontaneous diffusion of acetone in water creates an interfacial turbulence resulting in formation of particles in nanoscale. This principle was experimented for entrapment of Sb in PLGA. Pluronic F-127 (or poloxamer 407) was used as stabilizer. Pluronics are a group of ABA triblock copolymers having poly ethylene glycol (PEG) as outer block and hydrophobic poly (propylene oxide) (PPO) as middle block and can help in modifying the fate of synthesized nanoparticles [27]. Pluronic F-127 has also been approved recently by the US FDA for application in drug delivery devices. The new nano-device designed with Sb as a payload was studied in streptozotocin induced diabetic model as one remedial alternative in systemic hyperglycemia.

## Materials and Methods

### Materials

All solvents and water used were of HPLC grade (E. Merck, India). Silybin, streptozotocin, PLGA (50∶50, MW 40,000–75,000), chitosan (MW medium), dialysis tubing (MW cut off 12,400), fluorescein isothiocyanate (FITC), pluronic F-127 (MW 12,600) were all purchased from Sigma-Aldrich (St. Louis, MO, USA). Diagnostic kits for estimating blood glucose, cholesterol, triglyceride, aspartate aminotransferase (AST), alanine transaminase (ALT) and alkaline phosphatase (ALP) were purchased from Span Diagnostics Limited, India. Enzyme linked immunosorbent assay (ELISA) rat insulin kit was purchased from DRG Diagnostics, Germany. Glycohemoglobin estimation kit was purchased from EAGLE Diagnostics, USA. Windows Excel (version 2003; Redmond, WA), statistical package SPSS/10.0 (SPSS, USA), Sigmaplot (version 6.0; Zendal Scientific, USA) softwares were used for data analysis.

### Preparation of silybin nanoparticles (CSbnp)

Nanoparticles were prepared following a facile and scalable solvent diffusion technique. Briefly, 10 mg of Sb and 50 mg of PLGA were dissolved together in 3 mL of acetone. The organic phase was then added into a 30 mL of aqueous solution containing 1% w/v pluronic F-127. The addition was made by a syringe pump at a rate of 15 µL/sec under magnetic stirring. Stirring was continued for an additional period of 12 h to evaporate off acetone. Nanoparticles were then recovered by ultracentrifugation (Hitachi Koki, Japan) at 30,000 rpm for 30 min at 4°C. The particles were further washed two times with HPLC grade particle free water to remove unincorporated Sb, unbounded polymer and the stabilizers.

Final chitosan embossed silybin nanoparticles (CSbnp) were prepared by polyelectrolyte deposition of chitosan. Briefly, the nanoparticles prepared as above were dispersed in water and added drop wise into a 0.1% w/v chitosan solution in 1% v/v aqueous acetic acid under magnetic stirring for 2 h. CSbnps were harvested similarly by ultracentrifugation and subsequent two step washings.

### Characterization of Nanoparticles

#### Particle size, zeta potential and morphology

The particle size and size distribution of nanoparticles prepared were measured in Zetasizer Nano ZS **(**Malvern Instruments, Malvern, UK**)** against a 4 mw He–Ne laser beam with 633 nm wavelength at 25°C and back scattering angle of 173°. Aliquots from preparation batches were sampled in disposable cuvettes and the particle size along with polydispersity index (PDI) were recorded using appropriate parameter inputs. Zeta potential of sample sets was analyzed in the same instrument following the particle electrophoretic light scattering. Aliquots were injected in zeta cells and the zeta potential was determined under an electrical field.

The morphology and shapes of the nanoparticles were investigated under an atomic force microscope (AFM). Micrographs of CSbnp suspensions were obtained in tapping mode using RTESP tips having resonance frequency of 150–300 kHz at a scan speed of 1.2 Hz in a Pico plus 5500 ILM (Agilent, USA) atomic force microscope. Images were captured and analysed using Picoview 1.10.4 software.

Morphology and size of the nanoparticles were also evaluated by negative staining method in FEI Tecnai TM Transmission Electron Microscope (Netherland). For TEM measurements, 10 µL of CSbnp suspension in water was carefully placed on 300 mesh formvar-coated copper TEM grid (Ted Pella Inc., CA, USA) followed by staining with 2% w/v of uranyl acetate solution for 5 min. The excess solution on the grid was removed using a piece of fine filter paper and the samples were allowed to air dry for 10 h. Images were captured at 80 KV.

### FTIR spectroscopy

FTIR studies were carried out in a FT/IR-670 plus (Jasco, Tokyo, Japan) to detect interaction of each component before and after nanoparticulation. Sb, chitosan, PLGA, pluronic and the nanoparticles were pelletized individually with IR grade KBr in the ratio of 1∶100 in a hydraulic press at a pressure of 150 bar for 30 sec. The pellets were scanned over a range of 4000 to 400 cm^−1^ at a resolution of 4 cm^−1^ and the data stacked in Biorad KnowItAll software for analysis and overlap regions.

### Evaluation for silybin encapsulation efficiency

Encapsulation efficiency of CSbnp was determined from the amount of Sb originally taken and the amount remaining in the supernatant after harvesting the nanoparticles following a validated HPLC method [24]. Sample solutions from the preparation batch acetone solutions or the nanoparticle supernatants were filtered through a 0.22 µm membrane, diluted in HPLC mobile phase and injected into a HPLC system (Waters dual pump HPLC model 515, New Castle, DE). Sb was analyzed at 288 nm in a reverse phase C_18_ column (Supelco, Bellefonte, PA, USA) (25×4.6 mm, 5 µm size). Mobile phase used was 85% phosphoric acid: methanol : water (0.5∶46∶64 v/v/v) at a flow rate of 1 mL/min. Peak area (y) vs concentration (x) graph was first developed from different known concentrations of Sb and was used routinely.

### Chitosan coating estimation

Chitosan quintessence on CSbnp was measured using a chitosan electrostatic interaction reaction with alizarin red dye [28]. A standard graph from chitosan and alizarin reaction extinction recorded at 571 nm was used to estimate chitosan mass content both in the primary stock solution and in CSbnp preparation supernatant after centrifugation. The difference in mass was considered for estimation of chitosan coating. Experiments were run in triplicate in each case and the data recorded in percentage entrapment from three separate batch preparations.

### Silybin release kinetics and modeling

For drug release studies, CSbnp equivalent to 9 mg of Sb payload dispersed in 5 mL of phosphate buffer (100 mM, pH 7.4) was transferred into dialysis bags (MW cut off 12.4 KD) and was placed in glass vials containing 100 mL of buffer in a shaker bath maintained at 70 rpm, 37°C. At predetermined time intervals, 10 mL of phosphate buffer solution was removed for analysis and the release medium replaced with fresh buffer in order to maintain the sink conditions. The Sb mass released at definite time intervals was estimated in HPLC from the release medium aliquots [24]. Standard curve plot was used for analysis with necessary corrections for the dilution factors. The release data was fitted in Korsemeyer-Peppas model and the n and K values were calculated using sigma plot 6.0 software in order to understand the nanoparticle release mechanisms.

### CSbnp in streptozotocin (STZ) induced diabetic rats

#### Ethics Statements

Male Wistar rats weighing 170–200 g were procured from Central Research Institute, (Kolkata, India). Animals were acclimatized under standard laboratory conditions of relative humidity 50±10%, temperature 22±2°C and 12/12 light dark cycle for 2 weeks prior to the start of experiments. Access to water was *ad libitum* and standard pellet food (Hindustan Uniliver, India) supply was provided twice a day till the start of the experiments. This study was carried out in strict accordance with the recommendations in the Guide for the Committee for the Purpose of Control and Supervision of Experiments on Animals (CPCSEA), Government of India. The protocol was approved by the institutional animal ethical committee (IAEC) of University of Calcutta (Approval no. 506/01/a/CPCSEA/CUTech03).

### Induction of diabetes

Before the induction of diabetes, the animals were weighed and basal blood glucose level was measured. Glucose tolerance test (GTT) was carried out at the start of the experiment to assess the glucose homeostasis in normal conditions and to detect the stages of pre-diabetic condition if any. Animals were fasted for a period of eight hours prior to analysis with water *ad libitum*. Each test animal was then challenged intraperitoneally with a freshly prepared aqueous D-glucose solution (2.0 g/kg). Blood glucose level was measured in the blood samples taken from the tail vein after 30, 60, 90 and 120 min of glucose injection and also initially at the 0^th^ time. Individual data was recorded and animals with normal glucose homeostasis were considered for further experiments.

Diabetes was induced in overnight fasted groups by single intraperitoneal injection of STZ at a dose of 50 mg/kg body weight in freshly prepared citrate buffer (0.1 M pH 4.0) [21]. These animals were fed with standard pellet food and a 5% glucose solution *ad libitum*, for 72 h. Afterwards the glucose solution was replaced with water. The control animals received the vehicle alone. The diabetic state was assessed by measuring fasting glucose level of blood taken from the tail vein. Rats with a blood glucose level above 250 mg/dL were considered as diabetic and were used in further experiments.

### Study design

Animals were divided into four groups of 6 animals each with the following treatment schedule.

Group C – Normal control received normal saline only.
Group D – STZ only induced rats served as diabetic control.
Group SbT – STZ induced diabetic rats treated ip with Sb 50 mg/kg b.w for 28 days (diabetic and Sb treated).
Group CSbnpT – STZ induced diabetic rats treated ip with CSbnp equivalent to 50 mg/kg Sb payload for 28 days (diabetic and CSbnp treated).

### Blood glucose estimation

Fasting blood glucose (FBG) concentration was monitored in blood samples extracted from the tail of each animal every week during the entire experimental period and at the end of the treatment in the morning, using a glucometer (Dr. Morepen Gluco One Blood glucose monitoring system BG 03) with maximum measuring capacity of 600 mg/dL.

### Intraperitoneal glucose tolerance test (IPGTT)

IPGTT test was performed twenty four hours after the last dose of treatment following the procedure as discussed earlier for GTT. Briefly a sterile 20% glucose was injected (ip) at a dose of 2.0 g/kg body weight. Blood was collected from the tail vein to estimate glucose level before (0th time) and 30, 60, 90 and 120 min after glucose injection by glucometer.

### Euthanization and tissue preparations

After completion of IPGTT studies, animals were fasted overnight and FBG levels were checked. Body weight of each animal was recorded before and every week till the end of the 30 days experimental period. Blood samples (1.5–2.0 ml) were collected by cardiac puncture under light anesthesia. Samples were collected in marked vials added with or without anticoagulant for plasma and serum analysis and were stored at −20°C until further studies. The animals were finally euthanized by using CO_2_ gas and the tissue samples were collected for analysis. The liver and pancreas specimens were immediately removed and rinsed with chilled normal saline and dissected in two parts. The first part following a snap freeze in liquid nitrogen was stored at −80°C for further molecular and biochemical analyses and the second half was kept in 10% formalin for histopathological examinations.

### Biochemical assays

#### Serum analysis

The serum prepared from the blood samples was subjected to estimation of cholesterol and triglyceride levels by using estimation kits (Span Diagnostics Limited, India) following the manufacturer’s instructions.

Serum insulin levels were measured using rat insulin ELISA kit [29]. Metabolic enzyme activities of aspartate aminotransferase (AST), alanine transaminase (ALT) and alkaline phosphatase (ALP) were estimated using commercially available kits (Span Diagnostics Limited, India).

### Estimation of total protein

Total protein was estimated in serum and in liver tissue using bovine serum albumin (BSA) as a standard following the method of Lowry *et al.* [30].

### Serum fructosamine

Serum fructosamine (Amadori product) was determined by nitroblue tetrazolium (NBT) reduction assay according to the method of Johnson *et al*. [31]. Briefly, 1 mL of NBT reagent (0.5 mM NBT in 0.2 M sodium carbonate buffer pH 10.4) was added to serum (100 µL) and the mixture was incubated at 37°C for 1 h. The absorbance was measured at 530 nm against a reagent blank. The concentration of fructosamine was calculated compared to 1-deoxy-1-morpholino-fructose (1-DMF) as the standard [32].

### Estimation of oxidative stress markers and the antioxidant system

#### Determination of serum lipid peroxidation

Serum malondialdehyde (MDA) level as a measure of lipid peroxidation was assayed in the form of thiobarbituric acid (TBA) reactive substance following the method of Yagi [33]. Briefly, 400 µL of serum was diluted with 100 µl of distilled water. Trichloroacetic acid solution (TCA, 2.5 mL, 1.22 M) was added to the serum sample and the mixture was kept at room temperature for 15 min. 1.5 mL of 0.76% TBA containing 0.05 (M) NaOH was then added to the mixture and incubated in a boiling water bath for 30 min. The reaction mixture was cooled and 4 mL n-butanol was added into it. The resultant chromophore was extracted from the butanol phase. The generation of MDA was measured from the fluorescence emission intensity of the resultant chromogen at 553 nm by excitation at 515 nm. The results were expressed in fluorescence unit/µg of protein.

### Estimation of SOD, Catalase and GSH in liver tissue

Liver tissues were minced and homogenized (tissue homogenizer, TH 02, Omni International, Kennesaw, GA) in 10 mM potassium phosphate buffer containing 0.1 mM EDTA, pH 7.4, at a proportion of 1∶9 (w/v). The homogenate was centrifuged at 6000 g for 10 min at 4°C. The resultant supernatant was used for the determination of catalase and SOD activities and estimation of glutathione (GSH) content.

SOD activity was measured according to the method of Marklund and Marklund [34]. In this test, the degree of inhibition of pyrogallol auto-oxidation by the supernatant of the tissue homogenate (100 µL) was measured. One unit of enzyme activity was defined as the amount of enzyme necessary for inhibiting the reaction by 50%. The enzyme activity was expressed as units per gram of tissue.

The catalase assay was carried out as described by Aebi [35]. Briefly, 50 µL of the supernatant was taken with 2950 µL of phosphate buffer (10 mM, pH 7.4). Hydrogen peroxide (80 µL, 10 mM) was further added to initiate the reaction. A blank was prepared with 3000 µL of the phosphate buffer and 80 µL of H_2_O_2_ without tissue homogenates. The decrease in optical density due to decomposition of H_2_O_2_ was measured at the end of 1 min compared to the blank at 240 nm. Enzyme activity was defined in terms of units of catalase required to decompose 1 µM of H_2_O_2_ per minute at 25°C. The specific activity was expressed in terms of units/mg of tissue.

Tissue content of GSH was estimated following Ellman’s method with some modifications [36]. Briefly, 200 µL of sodium EDTA solution (20 mM) was added to 200 µL of the supernatant and was kept for 10 min at 4°C. 400 µL of 5% TCA was added and the mixture was left for 5 min at room temperature. After centrifugation, 500 µL of the supernatant was collected and 500 µL of Tris buffer (200 mM, pH 8.4) and 50 µL 5,5′-dithiobis-(2-nitrobenzoic acid) (DTNB, 100 mM) were added. Optical density was monitored after 3 min at 412 nm against deionized water. A blank reaction run was also run by mixing 500 µL Tris buffer and 50 µL DTNB and the average of absorbance at the beginning and at the end of the reaction was recorded. The blank reading was subtracted from each sample readings and the results were expressed as µM/mg of tissue.

### Determination of the glycogen content in liver

The glycogen content was estimated according to the method of Murat and Serfaty [37]. Briefly, liver tissues were homogenized in ice-cold citrate buffer (0.1 M, pH 4.2) at a ratio 1∶9 (w/v), followed by centrifugation at 10,600 g for 30 min at 4°C. The free glucose content in the supernatant was then measured by GOD/POD method using the assay kit (Span Diagnostics Limited, India) [38]. Amyloglucosidase (2 mg, Sigma, USA) was added to the homogenate (500 µL) and was further incubated for 4 h at 37°C. The total glucose content after incubation was then measured similarly. The glycogen content in the liver was calculated as the difference between total and free glucose.

### Estimation of glycohemoglobin (HbA_1c_) content

HbA_1c_ in blood was estimated by the ion-exchange resin method [39], [40]. Whole blood (100 µl) was mixed with lysing reagent and the hemolysate was loaded to a cation exchange column (EAGLE Diagnostics, USA). Unmodified hemoglobins were retained by the resin, and HbA_1c_ (fast fraction) was eluted. The percentage of HbA_1c_ was determined by measuring the ratio of absorbance of the HbA_1c_ fraction and the total hemoglobin fraction in the hemolysate at 415 nm.

### Advanced glycated end product (AGE)

RBC hemolysates were prepared after washing the cells with normal saline followed by hypotonic lysis in deionized water. Total hemoglobin (Hb) was isolated and purified by Sephadex G-100 column chromatography, pre-equilibrated with 50 mM potassium phosphate buffer, pH 7.4. The concentration of Hb was measured from Soret absorbance with the extinction coefficient as 125 mM^−1^cm^−1^ at 415 nm and the results were calculated on monomer basis [41]. AGEs in Hb were estimated spectrofluorimetrically from the fluorescence emission at 440 nm with excitation at 370 nm [42].

### Histopathological analysis

Tissues were fixed in 10% v/v formalin and dehydrated in a series of ethanol solutions (70, 80, 90, 100% v/v). The tissue samples were processed by using paraffin block techniques in wax. The samples were then sectioned (≅5 µ) stained with hematoxylin-eosin and mounted with neutral DPX medium. Photograph of stained sections were captured with a camera attached to a light microscope (B1 series, Motic, Xiamen, China).

### Statistical analysis

Data were expressed as mean ± SD. Gathered data were assessed using one-way analysis of variance (ANOVA) with *post hoc* pair-wise comparisons between groups using the Bonferroni method. For all analyses, P<0.05 was considered significant and P<0.001 highly significant. Statistical analysis was performed using the statistical package SPSS/10.0 (SPSS, USA).

## Results and Discussion

### Nanoparticles and characterizations

Nanoparticulation of biopharmaceutic class IV type compounds is one strategy that is helpful in dissolution improvements, specific administration and facilitated bioactivity [43]. PLGA nanocarriers with Sb payload were prepared by solvent diffusion of acetone in water environment. Diffusion of solvent in water phase resulted in rapid particle formation. As the solvent rapidly diffuses into the water phase, an interfacial turbulence results which, can cause deposition of polymer at the transient acetone/water interface. Some advantages of this technique are mild preparative conditions, avoidance of high stress force and an apparent ease in future scale ups. US FDA approved tri block polymer pluronic F-127 (or poloxamer 407) was used as a stabilizer in place of common agent PVA. In contrast to PVA, pluronics interact with both hydrophobic and hydrophilic domain and provide a brush-like coat which could provide stability to nanoparticles during preparation and in the physiological environment [44]. Chitosan association on nanoparticle surface was one likely reason for a sustained release and controlled drug permeability. Average hydrodynamic diameter of the prepared CSbnp was recorded in DLS as 229.7 nm and the PDI was 0.124. This indicated particle uniformity in the preparation system.

**Figure 1:**
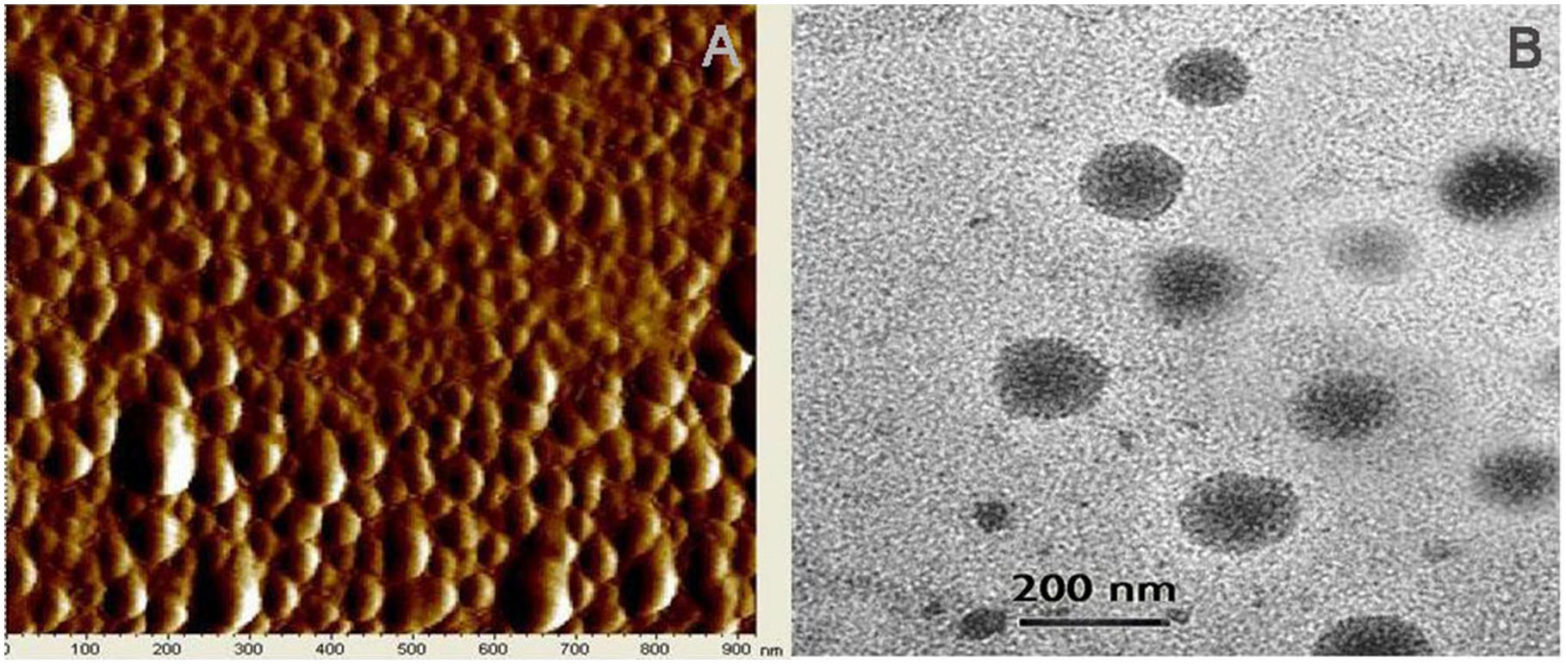
Shape and surface morphological of CSbnp. Atomic force microscopy of CSbnp (A) and transmission electron microscopy of CSbnp (B).

Presence of molecular layer of polycationic chitosan on PLGA was evident from a positive zeta potential value of +21 mV. Silybin entrapment percentage in CSbnp was determined routinely in a reverse phase HPLC [24] set up and was recorded as high as 92.11%. AFM analysis of CSbnp revealed smooth surface topography for particles with mostly spherical geometry (Figure 1). A degree of coalescence in the AFM scans over time was also noticed in the observation scales. Transmission electron microscopy showed discrete nanoparticles nearly spherical in shape having mean particle diameter of 184.6 nm. The diameter of the particles observed in the TEM was relatively smaller than the hydrodynamic diameters observed in the DLS method. Similar size differences were reported by other researchers [45].

**Figure 2:**
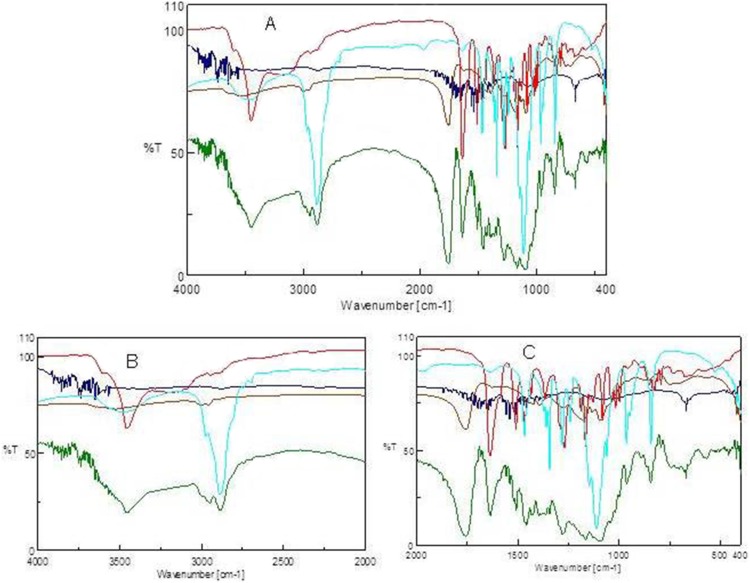
FTIR scans for silybin, PLGA, chitosan, pluoronic and CSbnp. A) FTIR scan over the mid IR region (cm^−1^), B) comparison zone upfield, C) comparison zone down field. [Colour codes silybin (red); PLGA (brown); chitosan (blue); pluoronic (sea green); CSbnp (green)].

In FTIR, the characteristic Sb benzopyran ring vibration was recorded at 1084 cm^−1^ [46] alongside the flavonolignan ketone response at 1634 cm^−1^. The C-H deformation was observed at 821 cm^−1^ and the aromatic ring stretching vibrations were at 1508 cm^−1^ (Figure 2). In case of PLGA, the ester -CO response was distinct at 1757 cm^−1^ while the biopolymer C-H stretching was recorded at 2997 cm^−1^. Chitosan when scanned in FTIR responded, at 1656 cm^−1^ and 1591 cm^−1^ due to amide I and amide II vibrations. In CSbnp, a strong shift due to electrostatic interaction of amide I appeared to a lower wave number at 1624 cm^−1^, and a feeble response for chitosan -NH was clear. Non-covalent conjugations were clearly attributed in case of CSbnp. Observations of amide I shift and the loss of amide II response due to strong electrostatic interactions between biopolymeric -COO^−^ and the chitosan NH_3_^+^ groups were thus confirmed [47].

### Chitosan estimation

Chitosan mass in final CSbnp was analyzed on the basis of free chitosan left in the supernatant initially and after particle separations. A standard curve y = 0.0037x+0.1649, R^2^ = 0.9863, originally developed from the concentration (x) vs. absorbance (y) due to chitosan alizarin reaction in acidic pH environment (pH 5) was used to quantify the chitosan mass. The percentage chitosan coating efficiency on weight basis was recorded as 77.81±3.23.

### In vitro release

**Figure 3:**
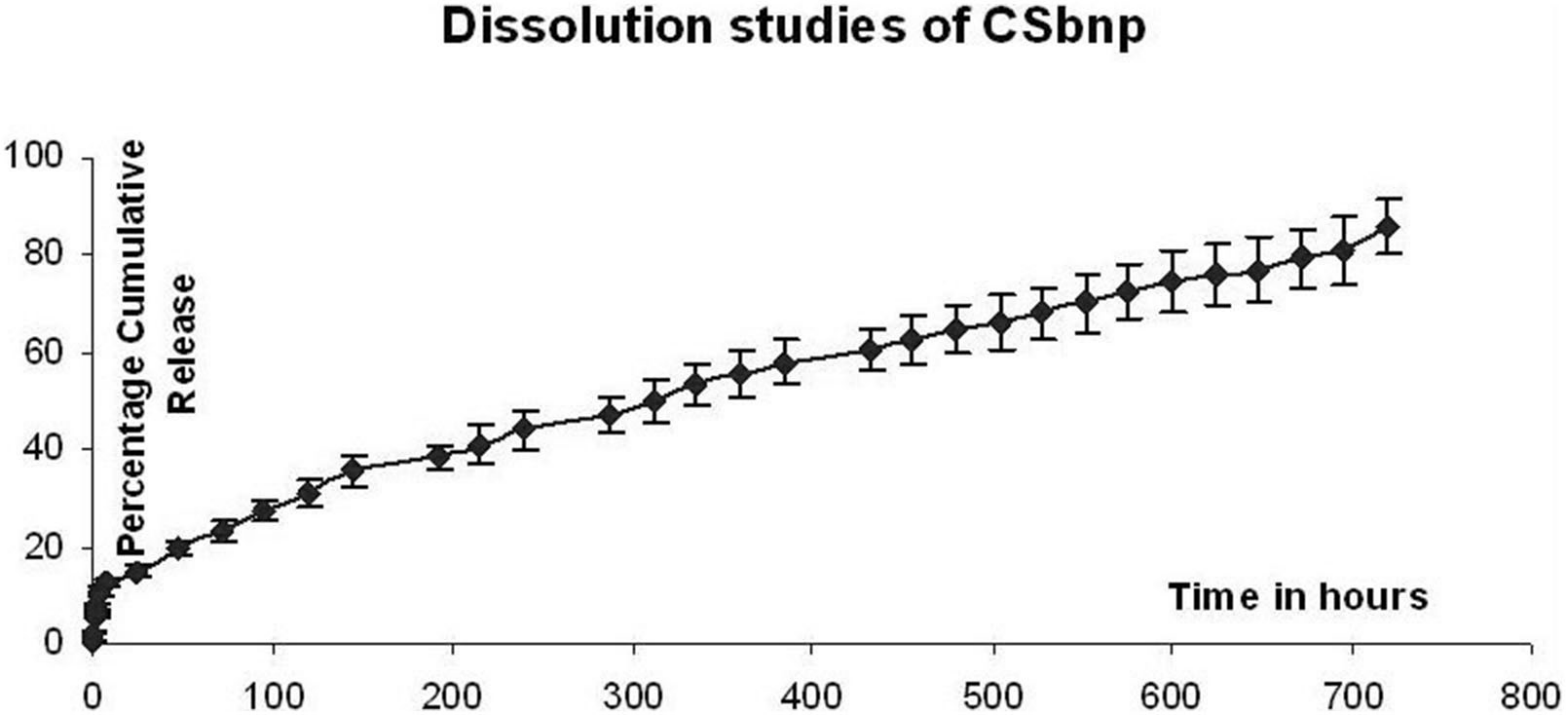
*In-vitro* dissolution studies for CSbnp. Results expressed as mean ± SD (n = 4). *In vitro* release of Sb from CSbnp was plotted as average percentage cumulative release over time.

Time-dependent cumulative Sb percentage release from CSbnp was studied and almost 85% of the initial Sb mass load was accountable during the study period (Figure 3). Release pattern was biphasic, initial fast release lasted up to 8 h followed by a sustained and steady release phase. Chitosan forms an entangled network layer on the particle surface which restricts the entry of water as well as prevents diffusion of drug molecules from nanoparticle surface to the surrounding medium that effectively controlled the release patterns [48]. Besides, the solubility of chitosan is pH dependent and at pH 7.4 it reduces the rate of diffusion of water in CSbnp. This initial fast release phase was followed by a very sustained and steady release for 31% of Sb mass payload over a period of 120 h. Drug release from particulate delivery devices is generally associated with intersects of multiple phenomenon including drug diffusion, polymer swelling, polymer erosion and degradation. The Peppas release exponent ‘n’ value of 0.45 for CSbnp indicated a potential overlapping of multiple incidents over the observation period [49].

### CSbnp in streptozotocin (STZ) induced diabetic rat

#### Effect of silybin and CSbnp on STZ induced hyperglycemia and glucose tolerance

**Figure 4:**
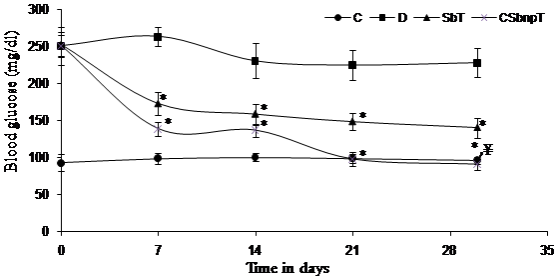
Blood glucose level in different groups during the 4 weeks experimental period. C – control group, D – diabetic group, SbT – Sb treated group, CSbnpT – CSbnp treated group. Results were expressed as mean ± SD (n = 6). *P<0.001 highly significant difference compared with diabetic group. ^¥^P<0.001 highly significant difference when compared between SbT and CSbnpT.

Fasting blood glucose was monitored in different groups of rats at different time intervals and is presented in Figure 4. STZ treatment caused a rise in blood glucose (P<0.001) in comparison to that in the control group. Both Sb and CSbnp treatment resulted in reduction of blood glucose level in comparison to the diabetic group D. However, CSbnp treatment exhibited a highly significant (P<0.001) down regulation of blood glucose levels compared to only Sb treated group by the end of the third week of the study period.

**Figure 5:**
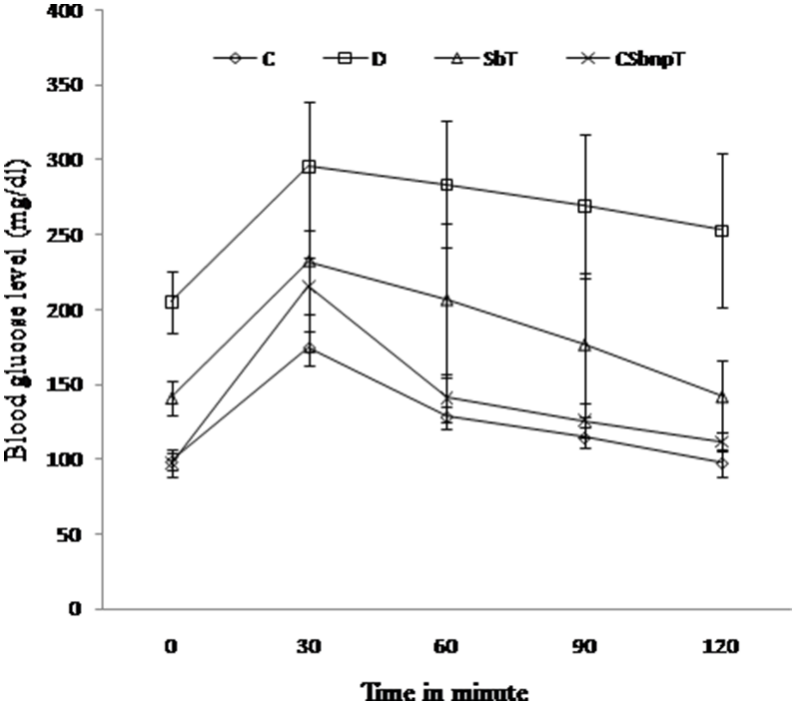
Intraperitoneal glucose tolerance test in different groups of rats. C – control group, D – diabetic group, SbT – Sb treated group, CSbnpT – CSbnp treated group. Data represented as mean ± SD (n = 6).

Glucose tolerance test is a more sensitive tool for recording early abnormalities in glucose regulation than fasting blood glucose measurement. Pancreatic dysfunction leads to defective utilization of glucose by the tissues that result in impaired glucose tolerance. Diabetic rats exhibited glucose intolerant behavior in comparison with the control (Figure 5). Rise of glucose level in diabetic rats was unabated even after two hours of glucose load due to impaired glucose homeostasis. Blood glucose level of diabetic rats when treated with CSbnp returned to near normal levels in 120 min after glucose injection. No significant difference was observed between glucose tolerance curves of CSbnpT group and the control group at the end of fourth week of treatment. Sb treated group (SbT) also exhibited a reversal effect but the blood glucose still remained higher than the normal level.

### Effect of Sb and CSbnp on body weight

**Figure 6:**
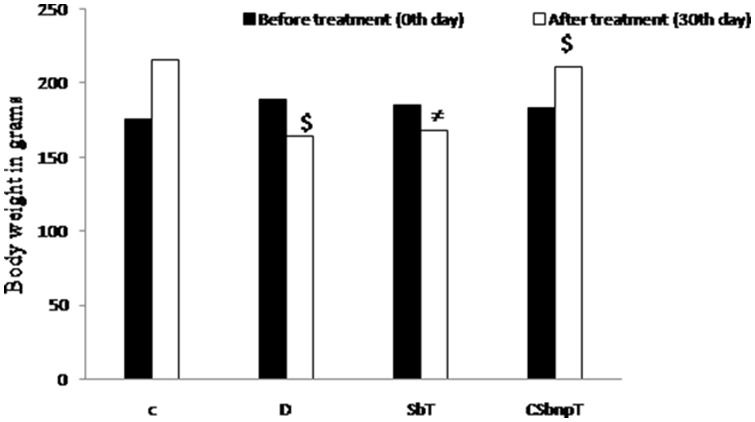
Effect of Sb and CSbnp treatment on body weight in rats. C – control group, D – diabetic group, SbT – Sb treated group, CSbnpT – CSbnp treated group. Results were expressed as mean ± SD (n = 6). ^≠^P<1.0 no significant difference compared with initial body weight. ^$^P<0.05 significant difference when compared with initial body.

The body weight recordings of different treatment groups before (0^th^ day) and after the experimental period are presented in Figure 6. Diabetic group of rats showed significant reduction in body weight (P<0.05) when compared with initial body weight. There was a significant gain in body weight in the diabetic rats treated with CSbnp (P<0.05). However there was no significant gain in body weight (P<1) of diabetic rats treated with Sb. The relative gain in body weight in CSbnp treated group compared to diabetic group revealed a steady recovery of animals from the prior diabetic conditions.

### Effect of Sb and CSbnp on serum insulin, cholesterol and triglyceride levels in diabetic rats

Induction of diabetes resulted in highly significant reduction in serum insulin level compared to that of control group (P<0.001) (Table 1). The level improved upon treatment with Sb, but the improvement was more pronounced when the group was treated with CSbnp. Administration of Sb produced moderate restoration of insulin level which was statistically insignificant in comparison to that of group D. However in case of CSbnpT group a marked improvement in insulin level (P<0.001) was observed. CSbnp treatment in diabetic rats may have assisted in insulin regeneration and the effect was also statistically highly significant (P<0.001) when compared to that of SbT animals.

**Table 1:** Effect of Sb and CSbnp on serum insulin, cholesterol and triglyceride levels in diabetic rats.

Animal group	Insulin (µg/lit)	Cholesterol (mg/dl)	Triglyceride (mg/dl)
**Control (C)**	0.60±0.06	77.6±6.54	55±8.02
**Diabetic (D)**	0.17±0.01	152.6±9.73	167.67±14.13
**D + Sb (SbT)**	0.29±0.06≠	108.2±10.12*	127.5±20.17φ
**D + CSbnp (CSbnpT)**	0.57±0.11* ^,^ ¥	80±6.13* ^,^ ¥	92.67±10.09* ^,^ ¥

Results expressed as mean ± SD (n = 6).

≠ P<1.0 no significant difference compared with D group.

*P<0.001 highly significant difference compared with D group.

¥ P<0.001 highly significant difference compared amongst Sb and CSbnp treated group.

φ P<0.01 significant difference compared with D group.

Both Sb and CSbnp treatment have significantly (P<0.001) reduced serum cholesterol levels as compared to that of the diabetic control group D. CSbnp reduces triglyceride level significantly (44.7%, P<0.001) in comparison to Sb (23.9%, P<0.01). Both serum cholesterol and triglyceride levels were significantly decreased in CSbnpT rats when compared to that in the diabetic group D indicating recovery. However, CSbnpT treated rats attained much lower values of blood cholesterol and triglyceride levels than that of SbT rats and the differences were of comparative statistical significance (P<0.001).

The increase in serum glucose and decrease in insulin of diabetic rats indicated the death of the pancreatic beta cells. However increase in serum insulin levels in SbT and CSbnpT groups indicated the probability of pancreatic β -cell regeneration in these groups. STZ-induced diabetes mellitus causes overproduction and decreased utilization of glucose by the tissues which results in disturbance of carbohydrate and fat metabolism and are characterized by hyperglycemia, hypertriglyceridemia, and hypercholesterolemia. Previous studies suggested that Sb in higher dosage reduced glucagon and induced stimulation of both gluconeogenesis and glycogenolysis resulting in down regulation of glucose 6-phosphate hydrolysis [20], [50]. Plasma levels of triglyceride, cholesterol and lipid are therefore likely decreased through the recovery of energy substrates, inhibition of lipid peroxidation, membrane stabilization and hyperglycemic depression [27].

### Liver marker enzyme analysis

Liver enzymes ALT, AST and ALP are responsible for proper functioning of the liver. Hyperglycemia induced hepatocellular damages lead to excessive leakage of these enzymes into the blood stream. A rise in AST and ALT concentrations in the serum indicated hepatocellular damages. ALP is one marker of biliary function and cholestasis. The increased levels of these enzymes in the diabetic rats were due to leakage from the liver cytosol as a result of hepatic tissue damages. STZ induced hyperglycemia established liver damage as evidenced by the elevated level of serum ALT, AST and ALP enzymes. Treatment with Sb and CSbnp helped in lowering of AST, ALT and ALP levels significantly when compared with that in group D (Table 2). Diabetic animals treated with Sb and CSbnp however showed differential activity on reduction of liver marker enzymes. The decreased AST, ALT and ALP levels of CSbnp treated group were statistically highly significant compared to that of only Sb treated group SbT (P<0.001). The significant reversal of AST, ALT and ALP levels in CSbnpT group indicated noticeable recovery due to CSbnp [27] treatment.

**Table 2:** Effect of Sb and CSbnp over liver metabolic enzymes in diabetic rats.

Animal group	AST (IU/L)	ALT (IU/L)	ALP (IU/L)
**Control (C)**	94±9.11	47.33±3.24	82.33±6.38
**Diabetic (D)**	168±11.57	90.33±7.18	213.66±17.86
**D + Sb (SbT)**	132.66±12.87φ	72.16±6.48*	188.33±13.54*
**D + CSbnp (CSbnpT)**	109.16±9.57* ^,^ ¥	58±6.65* ^,^ ¥	129.83±11.5* ^,^ ¥

Results expressed as mean ± SD (n = 6).

φ P<0.01 significant difference compared with D group.

*P<0.001 highly significant difference compared with D group.

¥ P<0.001 highly significant difference compared amongst Sb and CSbnp treated group.

### Effect of Sb and Csbnp on oxidative stress markers and antioxidant system in diabetic rats

#### Serum MDA estimation

**Figure 7:**
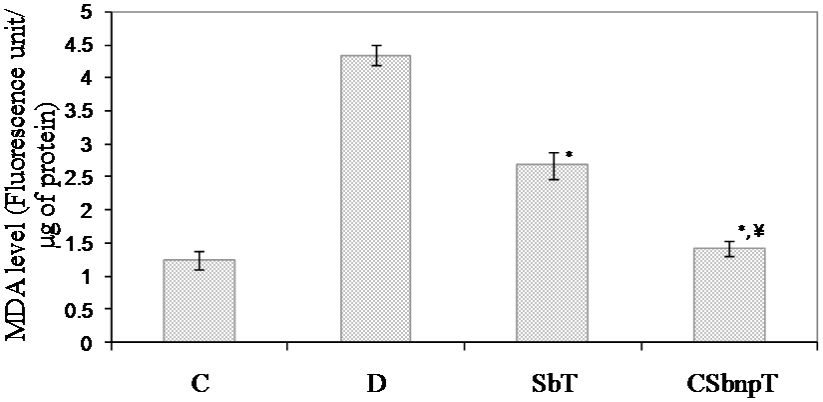
Effect of Sb and CSbnp treatment on serum MDA level in rats. C- control group, D – diabetic group, SbT – Sb treated group, CSbnpT – CSbnp treated group. Results were expressed as mean ± SD (n = 6). *P<0.001 highly significant difference compared with D group.^ ¥^P<0.001 highly significant difference compared amongst Sb and CSbnp treated group.

Oxidative stress in animals was measured by markers like secondary products of lipid peroxidation such as thiobarbituric acid reactive species (TBARS). Free radicals like CH_3_^+^ possess a very short half-life but affect bioactive molecular mechanisms specifically in diabetic conditions. Serum MDA level was measured from TBARS formation in different groups of rat following the method of Yagi [33]. Rise in MDA level was highly significant (P<0.001) in diabetic group with respect to that of the control group. Protective effects of Sb and CSbnp were evident from the significantly reduced levels of MDA in the respective treated groups. However decrease in MDA level of CSbnpT group was statistically significant when compared to that of only Sb treated group (P<0.001, Figure 7).

### Evaluation of antioxidant status

Oxidative stress and in particular reactive oxygen species (ROS) play an important role in induction of diabetes and its associated complications. An imbalance between reactive oxygen species (ROS) generation and the reduced activity of antioxidant defenses may lead to oxidative stress in diabetic condition [21]. Antioxidants could be preventive or protective in similar conditions and are precious in the treatment of diabetes.

Oxidative stress negatively affects the activities of catalase and SOD in the liver tissues and also raises the peroxidation levels [51]. Total antioxidant status was measured by the amount of enzymatic (SOD, catalase) and non enzymatic (GSH) markers and is presented in Table 3.

**Table 3:** Effect of Sb and CSbnp over antioxidant status of liver in diabetic rats.

Animal groups	SOD (U/gm of tissue)	Catalase (mU/mg of tissue)	GSH (µM/mg of tissue)
**Control (C)**	7.64±0.98	146.5±8.90	4.02±0.31
**Diabetic (D)**	3.42±0.64	88.5±8.76	2.54±0.21
**D + Sb (SbT)**	5.64±0.78≠	113.83±11.34φ	2.93±0.12€
**D + CSbnp (CSbnpT)**	6.84±0.32φ ^,^ ¥	134.16±8.74* ^,^ α	3.54±0.16* ^,^ ¥

Results expressed as mean ± SD (n = 6).

≠ P<1.0 no significant difference compared with D group.

φ P<0.01 significant difference compared with D group.

*P<0.001 highly significant difference compared with D group.

€ P<1.2 no significant difference compared with D group.

¥ P<0.001 highly significant difference compared amongst Sb and CSbnp treated group.

α P<0.05 significant difference compared amongst Sb and CSbnp treated group.

SOD catalyzes the conversion of the superoxide anion to hydrogen peroxide and the molecular oxygen species. The observed decrease in SOD activity in diabetic control rats could be due to inactivation by H_2_O_2_ or by glycosylation of the enzyme in diabetes [52]. Catalase is a haem containing ubiquitous enzyme which catalyzes the reduction of H_2_O_2_ and protects the tissues against reactive hydroxyl radicals. SOD and catalase activities were found to be significantly reduced (P<0.001) in diabetic rats (group D), as compared to those in control rats (group C). The observed decrease in SOD and catalase activities in diabetic rats might be due to inactivation of the enzymes by ROS or by glycosylation, as reported in diabetic condition [52], [53]. Sb treated group exhibited increased SOD level but was statistically insignificant while CSbnpT group showed significant increase in activities of antioxidant enzymes SOD (P<0.01) and catalase (p<0.001).

GSH is a direct scavenger of free radicals as well as a co-substrate for peroxide detoxification and also it has a versatile role in antioxidant defense. In diabetic control groups, the decreased GSH level may be due to its reduced synthesis or enhanced degradation by oxidative stress [54]. Restorative effect of Sb and CSbnp were indicated by the improved antioxidant status. However, compared to the Sb-treated group, CSbnp-treated rats demonstrated much closer SOD, catalase and GSH levels to those of the control group (Table 3).

Silymarin was reported to have protective effect on diabetes induced oxidative damages in the pancreas, kidney and liver [19], [55], [56]. The antioxidant effect of silymarin components is mainly due to its antiradical and reactive oxygen species scavenging capacities. Additionally polyphenolic silymarin compounds may play an important role in stabilizing lipid peroxidation due to their intrinsic reducing capacity [57].

### Estimation of glycogen content

**Figure 8:**
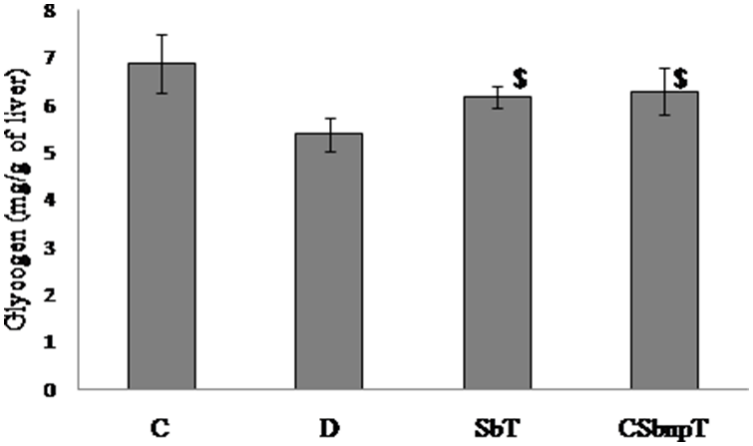
Effect of Sb and CSbnp on liver glycogen content. C- control group, D – diabetic group, SbT – Sb treated group, CSbnpT – CSbnp treated group. Results were expressed as mean ± SD (n = 6). ^$^P<0.05 significant difference compared with D group.

Excess glucose is converted into glycogen under the influence of insulin. Glycogen is considered as energy fuel of tissues especially in the liver and skeletal muscles. Glycogen contents measured in the liver tissues and are shown in Figure 8. Reduced glycogen level in group D may be related to insulin deficiency and significant improvement (P<0.05) of glycogen content in SbT and CSbnpT groups indicated the effective glucose utilization and storage in these groups. Maximum rise in liver glycogen content was however observed in CSbnp treated group that could be reasoned due to nano size assisted transport enhancement of Sb in liver.

### Estimation of serum fructosamine and HbA_1c_

Fructosamine is a ketoamine formed when the carbonyl group of glucose reacts with an amino group of proteins in protein glycation reaction [58]. The Amadori product or fructosamine is the first stable product of protein modification by glucose and its level in serum increases in diabetes [59]. Fructosamine can undergo oxidative cleavage resulting in the formation of advanced glycation end product (AGE). A significant rise in serum fructosamine level was observed in diabetic group in comparison to that of normal control (P<0.001) as shown in Table 4. Treatment with Sb and CSbnp caused significant reversal of fructosamine level with respect to diabetic group (P<0.001).

**Table 4:** Effect of Sb and CSbnp on glycohemoglobin (HbA_1c_) and fructosamine levels in diabetic rats.

Animal group	Serum fructosamine (µmol/µg of protein)	% of HbA_1c_
**Control (C)**	0.59±0.12	1.79±0.37
**Diabetic (D)**	2.72±0.64	5.88±0.58
**D + Sb (SbT)**	1.12±0.45*	4.33±0.12*
**D + CSbnp (CSbnpT)**	0.56±0.03*	2.24±0.24* ^,^ ¥

Results expressed as mean ± SD (n = 6).

*P<0.001 highly significant difference compared with D group.

¥ P<0.001 significant difference compared amongst Sb and CSbnp treated group.

Persistent hyperglycemia results in glycation of hemoglobin which leads to the formation of HbA_1c_, which is also proportional to blood glucose levels. The average HbA_1c_ level was found to be significantly higher in group D rats than that of control group C (P<0.001) indicating poor glycemic control. Diabetic rats treated with CSbnp showed a considerable reduction of glycation level of hemoglobin (P<0.001) compared to that of only Sb treated group indicating better glycemic control by nanoparticle treatment (Table 4).

### Estimation of AGEs

**Figure 9:**
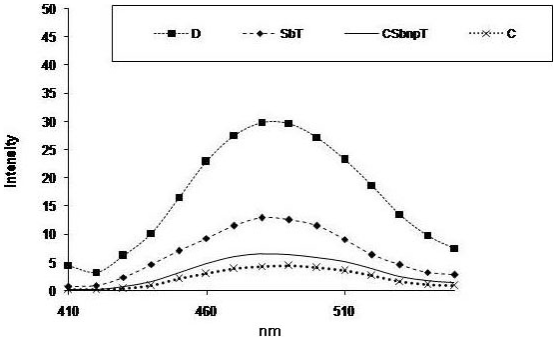
Effect of Sb and CSbnp on formation of advanced glycated end product (AGE). C- control group, D – diabetic group, SbT – Sb treated group, CSbnpT – CSbnp treated group.

In diabetes, Maillard reaction is common with the formation of AGEs, which are the root causes of different diabetic complications. AGEs in hemoglobin samples from different groups were estimated spectrofluorimetrically. Diabetic group showed increased presence of AGE in comparison to group C under fluorescence emission scan (Figure 9). Reduced level of AGE in hemoglobin was observed in case of CSbnpT group over the SbT group which might be related to directed Sb response against diabetes.

### Histopathological study

**Figure 10:**
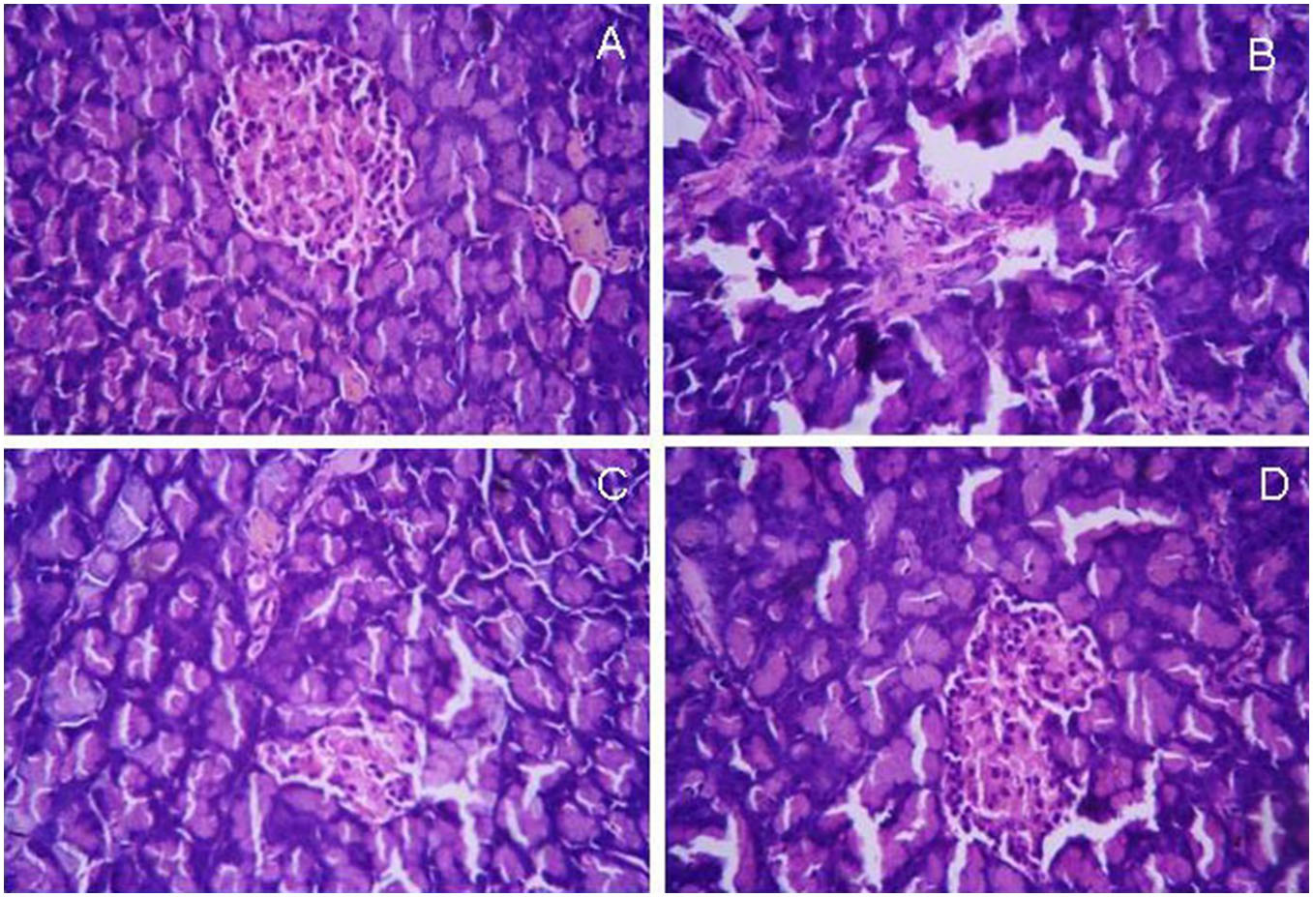
Histopathology examination of pancreas sections. (A) Normal Pancreas, (B) Diabetic Pancreas, (C) Sb treated pancreas, (C) CSbnp treated pancreas (Magnification 100×). Representative micrographs are shown.

Pancreatic sections of group C animals showed compact, regular, nearly rounded islet cells with well defined nuclei and a grainy appearance in the cytoplasm. (Figure 10A). No evidence of central necrosis was observed in normal pancreatic section. Histopathological analysis of pancreatic tissue slices in group D animals exhibited shrinkage of islet cells and a reduced number of islet cells. The peripheral islets are infiltrated with lymphocytes (Figure 10B).

Pancreatic tissue histoarchitecture of CSbnpT group recorded a marked improvement in the structural integrity of the islets of Langerhans when compared to the observations in group D. Islet histoarchitecture, the number of islet cells and peripheral islet infiltration of lymphocytes appeared to be considerably improved (Figure 10C). On the other hand, Sb treated group exhibited better histoarchitecture of islet cells with partial recovery in comparison to diabetic group (Figure 10D).

**Figure 11:**
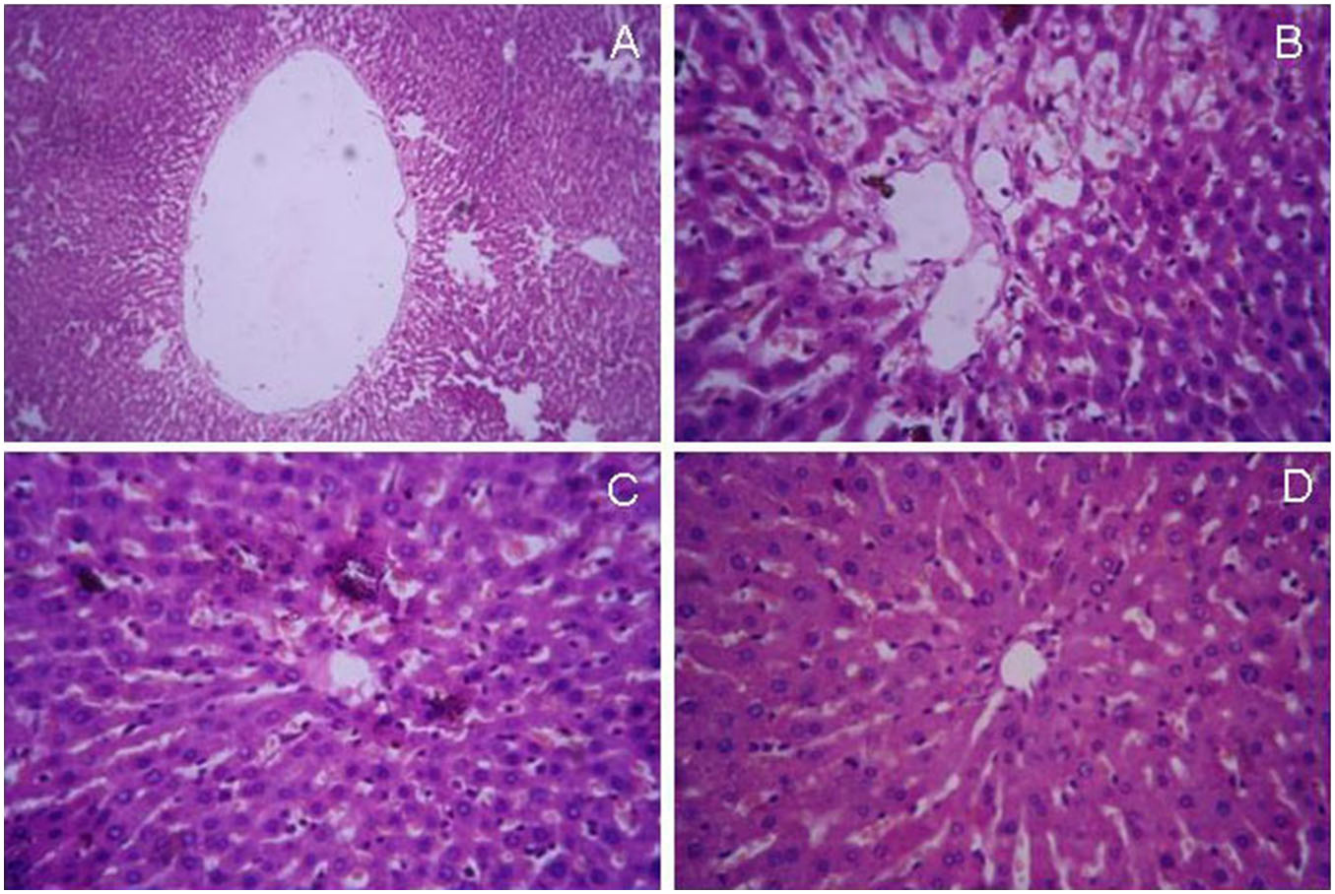
Histopathology examination of liver sections. (A) normal liver, (B) diabetic liver, (C) Sb treated liver (D) CSbnp treated liver (Magnification 100×). Representative micrographs are shown.

Liver sections of non-diabetic control animals exhibited regular hepatic architecture, prominent centrilobular vein, sinusoidal spaces and prominent nucleus (Figure 11A). In contrast, sections of the diabetic rat liver showed accumulation of fat droplets with distorted morphology of centrilobular vein, hepatocytes, and occurrence of sinusoidal dilatation (Figure 11B). Treatment with engineered nanoparticles stimulated significant revival of hepatic cytoarchitecture with reduction of fat droplets as well as sinusoidal abnormality (Figure 11D). The revival effect appeared to be better compared to that of Sb-administered animals (Figure 11C).

Streptozotocin is a nitrosourea analog which is known to accumulate selectively in pancreatic beta cells via the low-affinity glucose transporter-2 (GLUT2) in the plasma membrane [60]. Selective toxicity of this agent causes DNA alkylation, protein glycosylation and depletion of ATP leading to beta cell damages [61]–[63]. Insulin biosynthesis, glucose-induced insulin secretion and glucose metabolism get affected as a result of impaired beta cell functions [64], [65]. Hyperglycemic conditions in diabetes leads to free radicals generation and induces oxidative stress by activating mitochondrial NADPH oxidase [66], [67]. Oxidative stress conditions prevent insulin transport through endothelial walls and limits delivery of the hormone to the tissues [56].

Sm is reported to be associated with strong antioxidant properties, regenerative effect on beta cells, stabilizing effect on cell membrane and enhancement of membrane permeability to glucose [68]–[70]. Studies have also demonstrated that Sb could protect pancreas from cyclosporine A-induced toxicity and effect potent hypoglycemic effects in both type 1 and type 2 diabetes mellitus [64], [16]. Sb also dose-dependently reduces the glucagon-induced stimulation of both gluconeogenesis and glycogenolysis and induces a potent decrease in glucose-6- phosphate hydrolysis [20], [50]. Sb induced stabilization of mitochondrial membrane potential and inhibition of cytokines production likely aid in antioxidant efficacy.

The results of the present study showed that administration of Sb exhibited an overall beneficial effect on streptozotocin induced diabetes in rats. Biopharmaceutic enhancement of Sb through nanoparticle engineering was marked by down regulation of blood glucose within two weeks of treatment. Sb also lowered blood glucose level but failed to normalize the hyperglycemic condition in contrast to the animals treated with CSbnp nanoparticles. CSbnp treatment exhibited normoglycemic conditions rapidly after three weeks of treatment. Nanoparticulation of Sb favored size assisted passive transport [71], improved dissolution and enhanced cellular uptake which resulted in improved efficacy of CSbnp in diabetic animals. Regulated release of Sb could also be one reason for decreased gluconeogenesis in CSbnpT group which was reflected in the higher liver glycogen content in treated animals. Besides CSbnp induced improved antioxidant defense has helped to regenerate pancreatic beta cell population consequently affecting in higher insulin blood levels. Engineered nanoformulations like CSbnp with low toxic therapeutic pay load thus could be a significant contributor in early management of diabetes conditions.

## Conclusion

Silybin, the principle bio-active component of silymarin was proposed earlier as an alternative therapy in diabetes. Silybin was successfully nanoparticulated following facile particle engineering techniques. The molecular loading was higher at 92.11% and the release profile in new engineered nanoparticles CSbnp was sustained. CSbnp contribution in diabetes control was profound and a remarkable response was recorded in the test animals with recovery to nearly normal insulin levels and reduced glycated hemoglobin parameters. Liver concentric effects of CSbnp could be one reason that facilitated antioxidant defence mechanism and aided in beta cell regeneration. This affected in increased insulin availability and a concomitant protection for liver glycogen stores. Such conditions are also likely due to Sb induced interference in neoglucogenesis pathways. The designed Sb nanomedicines therefore provided newer directions in diabetes management. The nanoparticles are likely to aid further in diabetic control and serve as an adjunct therapeutic.
